# Evaluation of a multiphasic parasite clearance profile after treatment of experimental human infection with the investigational anti-malarial M5717 using segmented mixed effect models

**DOI:** 10.1186/s12936-023-04627-x

**Published:** 2023-06-28

**Authors:** Xiaoyan Yin, Ying Li, Wilhelmina Bagchus, Özkan Yalkinoglu, Deon Bezuidenhout, Aliona Tappert, James McCarthy, Louise Marquart, Claude Oeuvray

**Affiliations:** 1 EMD Serono Research & Development Institute, Inc. (an Affiliate of Merck KGaA), 45 Middlesex Turnpike, Billerica, MA 01821 USA; 2 WMB: Merck Serono S.A (an Affiliate of Merck KGaA), Lausanne, Switzerland; 3 Merck KGaA, Darmstadt, Germany; 4 QIMR Berghofer Medical Research Institute, Brisbane, QLD Australia; 5 The Global Health Institute of Merck (an Affiliate of Merck KGaA), Eysins, Switzerland; 6 Present Address: The Peter Doherty Institute for Infection and Immunity, The University of Melbourne and the Royal Melbourne Hospital, Melbourne, VIC Australia; 7 Present Address: School of Public Health, University of Queensland, Brisbane, Australia

**Keywords:** Parasite clearance, Segmented mixed model, Malaria

## Abstract

**Background:**

Evaluation of parasite clearance patterns in experimental human infection trials helps increase understanding of drug action. In a previously reported phase Ib trial of a new investigational anti-malarial drug M5717, parasite clearance showed a biphasic linear pattern: slow removal phase with a near flat clearance rate followed by a fast clearance phase with a steep slope. In this study three statistical approaches were implemented and compared to estimate the parasite clearance rate for each phase and the time point corresponding to the change of clearance rates (changepoint between the two phases).

**Methods:**

Data using three M5717 doses 150 mg (n = 6), 400 mg (n = 8), 800 mg (n = 8) were used to estimate biphasic clearance rates. Three models were investigated: firstly, segmented mixed models with estimated changepoint—models with/without random effects in various parameters were compared. Secondly, a segmented mixed model using grid search—this method is similar to the first except that changepoints were not estimated, instead they were selected based on model fit from given candidate values. Thirdly, a two-stage approach whereby a segmented regression model fit to each participant followed by a meta-analysis method. Hourly rate of parasite clearance (HRPC) interpreted as the percentage of parasites removed each hour was calculated.

**Results:**

The three models generated similar results. Using segmented mixed models, the estimated changepoints after treatment in hours (95% CI) were: 150 mg: 33.9 (28.7, 39.1); 400 mg: 57.4 (52.5, 62.4); and 800 mg: 52.8 (47.4, 58.1). For all three treatment groups, there was nearly no clearance before the changepoints, but rapid clearance in the second phase (HRPC [95% CI]): 150 mg: 16.8% (14.3, 19.1%); 400 mg: 18.6% (16.0, 21.1%); and 800 mg: 11.7% (9.3, 14.1%).

**Conclusions:**

All three statistical approaches are effective tools to characterize the bi-phasic clearance of M5717 in the phase 1b experimental *Plasmodium falciparum* malaria human infection study. The statistical approaches produced similar results to estimate the two-phase clearance rates and the changepoint for each treatment dose of M5717. However, the segmented mixed model with random changepoints has several advantages: it is computationally efficient, provides precision for changepoint estimates and is robust concerning outlying datapoints or individuals.

**Supplementary Information:**

The online version contains supplementary material available at 10.1186/s12936-023-04627-x.

## Background

Globally, malaria continues to bear a huge disease burden, and in 2020 alone affected 241 million people, resulting in 627,000 deaths [1]. The pharmaceutical industry, in partnership with Product Development Partners and the World Health Organization (WHO), is engaged in developing drugs to eliminate malaria. In early-stage clinical trials, it is critical for developers and investigators to understand how new anti-parasitic drugs work in humans. Key information is obtained by evaluating patterns of parasite clearance after drug treatment. Human experimental challenge models (a safe and reliable approach of inducing controlled blood stage *Plasmodium falciparum* malaria infection in healthy volunteers) [2, 3], have been developed and standardized for malaria to allow for early readout of parasite clearance.

A frequently used challenge model is induced blood-stage malaria (IBSM), which involves intravenously inoculating healthy volunteers with synchronous *P. falciparum* ring-stage parasites. The volunteers are monitored for safety, and once the parasite count reaches a certain threshold, the volunteers are treated with the drug of interest. Drug concentration and parasite counts are measured over time and can be used to analyse pharmacokinetic-pharmacodynamics (PK-PD)[4]. The life cycle of malaria in human body and its clearance mechanism have previously been described in detail [5]. As indicated in the work by White [5], anti-malarial drug effects on parasite clearance demonstrated through PK-PD modelling can be a useful tool in predicting therapeutic responses and dose-finding. Marquart et al. [6], Flegg et al. [7], Jamsen et al*.* [8] and Sharifi-Malvajerdi et al*.* [9] have analysed the pharmacodynamic effect of anti-malarial treatments based on controlled human malaria infection models and quantitative polymerase chain reaction (qPCR), and estimated the parasite reduction ratio and parasite clearance half-lives.

A phase I clinical trial sponsored by Merck KGaA, Darmstadt, Germany, describes the anti-malarial activities of a new investigational drug, M5717, using controlled infection of malaria in humans, specifically, by applying the IBSM challenge model [10]. A biphasic linear pattern in parasite clearance was observed in study participants after M5717 treatment. Specifically, there was a slow parasite clearance phase with a near zero clearance rate before a fast parasite clearance phase with a steep clearance rate. The biphasic clearance profile is specific to M5717 and attributed to the mode of action of the molecule, stopping parasite protein synthesis through inhibition of the *P. falciparum* elongation factor 2 enzyme.

To estimate the bi-phasic parasite clearance rate, the timing of the changepoints (an instance in time where the statistical properties before and after this time point differ), and their dependence on treatment groups of M5717, an appropriate statistical analysis method is required. This method needs to manage two properties of the data: repeated measurements which are correlated within individuals, and a segmented pattern with changepoint(s).

In the original analysis of the phase 1b study [11], estimation of the bi-phasic clearance was based on a two-stage approach. Firstly, the clearance rates and the changepoint were estimated for each individual using segmented regression [12]. Secondly, a combined estimate of the clearance rates and changepoint were calculated as a weighted average across all individuals for each parameter (using the inverse variance as the weight). However, this approach has some disadvantages: Firstly, fitting one regression model for each individual does not use the data efficiently because the estimate of variability in model residuals is not shared between individuals. Secondly, there is no measure of the overall fit to evaluate the performance of the model, which may be necessary to determine whether to include certain covariates or interactions in the model. Thirdly, at individual level the data can be sparse under some circumstances, and the fitted regression model may be unstable or unreliable.

The most widely used conventional method for analysing repeated measurement data is the mixed-effects model [13] in which a random effect(s) is assigned at the individual level to account for within individual correlations. Most statistical packages have well-developed procedures or functions to implement linear or generalized linear regression models with mixed effects. Mixed models do not naturally accommodate for multiple phases in the slopes i.e. segments, but if the changepoints are known, a relatively simple manipulation in the dataset can make this accommodation. Huang [14] demonstrated a piecewise (i.e. segmented) linear mixed model using statistical analysis system (SAS) in which the changepoint or breakpoint was required to be known or chosen in advance. The challenge in analysing data from the Phase 1b study is that the changepoints in the trajectories of parasite clearance are unknown. Modelling the changepoint itself, in a Bayesian [15] or likelihood framework [12] is commonly practiced in many areas, but mostly in the context of non-correlated data analysis [16].

Therefore, a mixed effects model that allows changepoints to be estimated and potential adjustment for covariates can provide a flexible modelling approach. A segmented mixed model with random changepoints in a likelihood-based framework has been developed [17], which allows for covariates and random effects for all model parameters including changepoints. This model does not require a smooth or parametric transition between segments, and allows for a linear or non-linear segment making it ideal for analysing the M5717 parasite clearance data. Recently this method has been implemented to model blood pressure trajectories in a population study [18]. The segmented mixed model with random changepoints has not yet been used to estimate bi-phasic parasite clearance rates, though a similar Bayesian method has been developed and used to estimate different parasite clearance rates with lag and tail phases [9, 19].

In this study, we implement three statistical methods to estimate parasite clearance rates for the 22 participants administered with M5717 in the Phase 1b trial. Firstly, the segmented mixed model with random changepoints [17] was employed. Using data across all participants, this method allowed simultaneous estimation of the slopes and changepoints for the three M5717 treatment groups whilst allowing for random effects of intercepts, slopes and changepoints. Secondly, a grid search method for changepoint based on segmented mixed models with two slopes was explored following the tutorial of Huang [14]. This method requires performing many segmented mixed models, whereby for each model the changepoint is considered known and presumed to be one of many candidate values on the time axis for each treatment group. For each set of possible changepoints, a segmented model was performed and a model fit statistic calculated. The model with best fit was chosen and used for determining the changepoints and estimating clearance rates. Thirdly, the two-stage approach of fitting a segmented regression model [12] for each participant followed by meta-analysis was implemented. Using each method, the parasite clearance rates for the two phases was estimated and compared and the timing of changepoints was estimated for each of the three M5717 treatment groups.

## Methods

### Study design, inoculation and treatment

As the study has been previously reported [10, 11], the clinical trial and data analysed are not described in detail. In brief, healthy volunteers were inoculated intravenously with erythrocytes harboring a defined number of *P. falciparum* parasites. The evolution of the parasitaemia was measured by quantitative polymerase chain reaction (qPCR) [20] over time, before and after single dose treatment with M5717.

Three treatment groups of participants were enrolled to test different doses of M5717: 150 mg (n = 6), 400 mg (n = 8), 800 mg (n = 8). Participants were evaluated for the presence of a patent parasitaemia by qPCR once per day from 4 days after inoculation, then once detected, twice daily until M5717 administration at the trial site which was about 9 days after inoculation (when the parasitaemia was above 5,000 parasites/mL). After treatment (Day 1, hour 0), the participants continued to be evaluated twice daily or more intensively until clearance of parasites (PCR negative) or up to 144 h after treatment, whichever came first. Thereafter, visits were scheduled daily or twice daily until treatment threshold was reached and then every few days to monitor recrudescence up to day 44 [11]. On Day 22, all participants were given rescue medication with artemether-lumefantrine and primaquine to ensure final clearance. If parasitaemia failed to clear or recrudescence occurred, rescue treatment was administered early. The study objectives were to establish parasite clearance profiles after treatment, and to understand their relationship with M5717 doses [11].

The objective of this analysis was to estimate the slope(s) and changepoints (if any) used to estimate the clearance profiles. This analysis used log_10_ transformed geometric mean of the triplicate parasitaemia data measured from M5717 administration (0 h) to PCR negative (all triplicates not-detected) up to 144 h after treatment. No recrudescence occurred within this period. The raw data for each participant appeared to have two log-linear clearance phases, hence the parameters of interest were the two slopes and the changepoint where the two slopes transit.

### Modelling methods

For the following description of the models, $y_{ij}$ was denoted to be the parasitaemia measurement for the $i^{th}$ individual ($i = 1, \ldots ,N$) at time $t_{ij} \left( {j = 1, \ldots ,n_{i} } \right)$, where $t_{i1} , \ldots , t_{{in_{i} }}$ was the time after treatment administration in hours for the $i^{th}$ individual. The following methods were used to calculate the clearance rate, and hourly rate of parasite clearance (HRPC), which was defined as the percentage of parasites cleared in each hour. For each clearance stage HRPC was calculated as the $1 - 10^{slope}$. Other common measures such as parasite clearance half-life and parasite reduction ratio [6] were also derived from the same regression coefficient. Additional details are provided in Supplementary Material: Additional file 1.

### The segmented mixed effects model with random changepoints

To account for the change-point in the two parasite clearance rates, $\psi_{i}$ was denoted to be the time of the change-point between the two clearance phases for the $i^{th}$ individual. The segmented mixed effects model with random changepoints [17] was given by:

1 $$y_{ij} = \beta_{0i} + \beta_{1i} t_{ij} + \delta_{i} \left( {t_{ij} - \psi_{i} } \right)I\left( {t_{ij} > \psi_{i} } \right) + \varepsilon_{ij}$$

where participant $\beta_{0i}$, $\beta_{1i}$, $\delta_{i}$ and $\varepsilon_{i}$ were the intercept, slope 1, the difference between slope 1 and slope 2, and the error term, respectively, for individual $i$, and $I\left( . \right)$ was an indicator function which takes value 1 if a data point was after the changepoint ($\psi_{i}$) and 0 otherwise. Each of the parameters $\beta_{0i}$, $\beta_{1i}$, $\psi_{i}$ and $\delta_{i}$ were the sum of a fixed effect and a random effect, e.g. $\delta_{i} = \delta + d_{i} ,$ and the fixed term could depend on covariates, e.g. $\beta_{1i} = \beta_{10} + x_{1i}^{T} \beta_{1} + b_{1i}$. The changepoint parameter composition when covariates were included was more complicated, but had a similar style, as detailed in Muggeo et al*.* [17], which could be implemented in R [21].

In this analysis, the outcome variable was log_10_(parasitaemia/mL). All models were adjusted for baseline parasite levels, i.e. the log_10_(parasitaemia/mL) at the time of treatment, and treatment group assuming post-treatment parasite level depended on the initial parasite level and treatment dose in addition to time. A random intercept for each individual, $\beta_{0i}$, was included in all the models. Treatment group was used as a categorical variable as the dose–response relationship was not assumed to be linear. A total of four models with different fixed and random effects were considered and are detailed in the Supplementary Material: Additional file 2. Briefly, Model 1 (M1) had fixed and same slopes for all treatment groups, fixed and same changepoint for all treatment groups. Model 2 (M2) had fixed slopes that differed for treatment groups, fixed changepoint that differed for treatment groups. Model 3 (M3) built on M2 by including random slopes for each individual and Model 4 (M4) built on M2 by including random effects for the slopes and also for the changepoints. As shown in the Supplementary Material: Additional file 3, M4 showed best fit to the data indicating that random effects for all the slope and changepoint parameters are necessary. Hence in the next two segmented mixed effects model methods, we used the models that are similar to M4 in structure.

### Segmented mixed effects model with grid search for changepoints

Instead of estimating changepoints directly as detailed in the previous section, the segmented mixed effects model with grid-search method treats changepoints in a non-parametric way. It was assumed the changepoints could be any reasonable timepoint on the time axis within the study duration based on the observed data. For each set of possible changepoints (i.e. one for each treatment group), a segmented mixed model was implemented and the fit statistic Akaike information criterion (AIC) was calculated. By comparing the model fits given different sets of changepoints, the best fit (i.e. changepoint corresponding to the minimum AIC) was selected as the final model, which provided estimates of slopes.

**Fig. 1:**
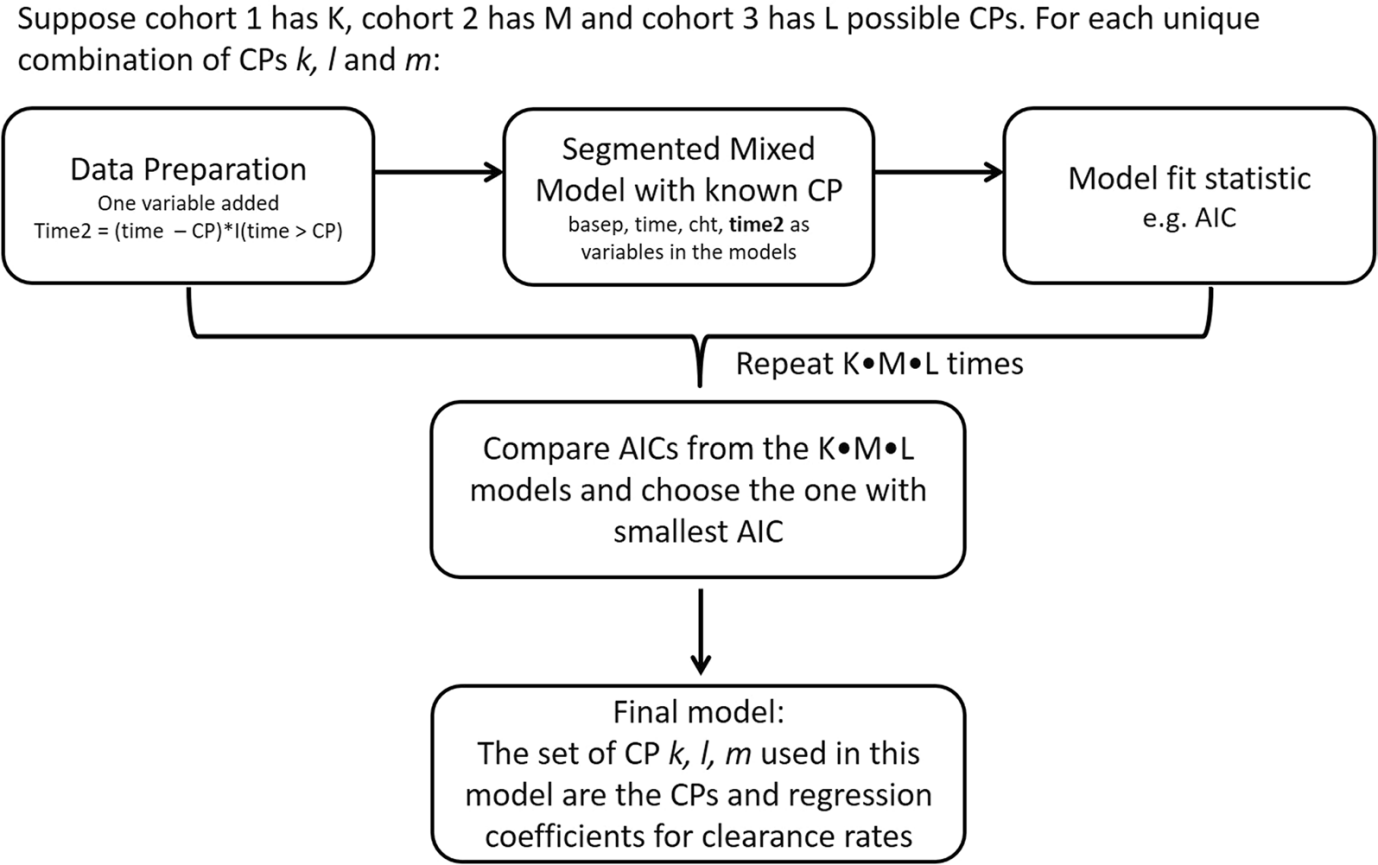
Flowchart for changepoint (CP) identification based on grid search

The process is illustrated in the flow chart (Fig. 1). Each reatment group was assumed to have K, M, L candidate changepoints (150, 400 and 800 mg group, respectively), thus in total K $\times$ M $\times$ L unique combinations (or sets) of candidate changepoints. For each set of candidate changepoints, at the data preparation step, one more time variable *time2* was created for each participant: before the changepoint its value was 0, and after the changepoint the value was the actual time minus the changepoint value.

Changepoints hence were “known” values in each round. Then at the segmented mixed model step, both *time* and *time2* were used as explanatory variables in the same model, so that the corresponding coefficients were the slope 1 and the difference between slope 1 and slope 2. The corresponding model fit statistic AIC was provided. This whole process was repeated for K $\times$ M $\times$ L times providing K $\times$ M $\times$ L AIC values. Ultimately, the model with the smallest AIC was chosen as the final model. The corresponding *k*, *m*, *l* used and the regression coefficients estimated in this chosen model were the identified changepoints and slope estimates for the three treatment groups, respectively. In this analysis, the changepoints at all integer values between 25 and 60 for each of *K*, *M* and *L* were tested. The segmented mixed model was set the same as M4 above except that changepoint was not a parameter to be estimated. Instead *time2* was included as an additional regressor. Compound symmetry structure was used for all random effect covariance matrices. This analysis was performed using nlme package in R [22].

In these models, *basep* was the variable name for baseline parasite level, log_10_(# parasites) at hour 0; *cht* is the variables for cohort. *time* was the time variable and its coefficient was the slope 1 for Cohort 3; CP: changepoint.

### Two-stage segmented regression approach

Unlike the two methods described above that modelled data from all individuals simultaneously, in the two-stage approach, a unique segmented regression model [12] was fit to the parasitaemia measurements of each participant; then the model parameter estimates across individuals were combined using a meta-analysis approach.

#### Step 1: Segmented model at individual level

For a given participant, to model the relationship of the parasitaemia over time, the segmented regression model (Eq. 2) had a similar form as the segmented mixed model (Eq. 1) without the random effects:

2 $$y_{j} = \beta_{0} + \beta_{1} t_{j} + \delta \left( {t_{j} - \psi } \right)I\left( {t_{j} > \psi } \right) + \varepsilon_{j}$$

where $y_{j}$ is the *j*^*th*^ parasitaemia measurement, *t* was the time since treatment administration in hours, $\beta_{0}$, $\beta_{1}$, $\delta$ and $\varepsilon_{j}$ were the intercept, slope 1, the difference between slope 1 and slope 2, and the error term, respectively, and $I\left( . \right)$ was an indicator function which takes value 1 if a data point is after the changepoint ($\psi$) and 0 otherwise. The model resembled simple linear regression except it had two slopes that transited at the changepoint. The estimation of the changepoint was implemented in R using the segmented package [23]. The process of segmenting the function involved an iterative procedure with bootstrap sampling to identify a changepoint that split the function into two slopes. The iterative procedure stopped when the change in slope difference estimate was not significantly different from 0. An initial estimate of the changepoint was needed to help with model convergence. The participant specific estimates including for parameters $\beta_{1}$, $\delta$ and $\psi$ from the converged final model were saved for meta-analysis.

#### Step 2: Meta-analysis

For each treatment group, for each of the parameters $\beta_{1}$, $\delta$ and $\psi$, the pooled estimate was calculated across all individuals using meta-analysis with random effects techniques [24]. The random effect in the meta-analysis assumed the parameter of interest from different individuals followed a normal distribution rather than being identical. These pooled clearance rate estimates were converted to HRPC.

Of note, in the primary trial publication [11] the corresponding results were obtained using this method. Two participants were excluded from the meta-analysis for the 400 mg because of their outlying estimated breakpoints and insufficient points in the second phase, and one individual was excluded from the meta-analysis for the 800 mg dose due to very low parasitaemia throughout the study and imprecise estimates of the segmented regression. Details about this exclusion are in the study manuscript. For this methodology comparison study, no participant was excluded from analyses.

All the analyses were performed using R version 3.6.1 (R Core Team [2019]).

### Ethics approval and consent to participate

This study was approved by the QIMR Berghofer Human Research Ethics Committee. Ethics number P2334/QP16C19. All study volunteers were required to sign an informed consent prior to entry onto the study.

## Results

The characteristics of the study participants are in the primary trial publication [11]. In brief, these healthy volunteers were all males, mean age 28 (SD 9) years old, mostly white (73%) and non-Hispanic or Latino (86%).

**Fig. 2:**
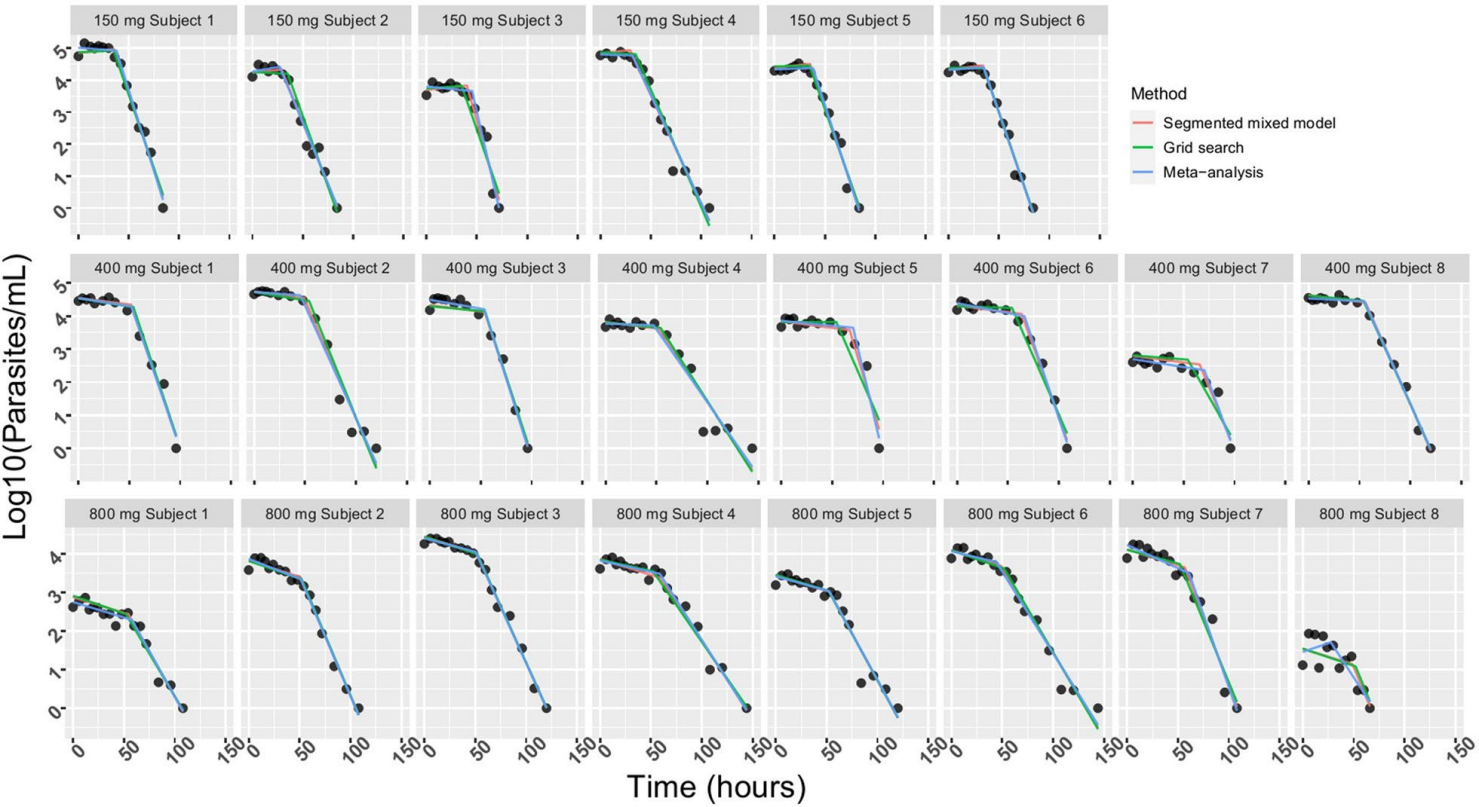
Fitted parasite clearance profile for all participants from M4, all estimation methods. Each panel depicts one participant, aligned by treatment group. Dots represents observed data, and lines are for fitted model

All three statistical methods to estimate the bi-phasic parasite clearance profile of M5717 performed similarly. Table 1 has the parameter estimates for all three methods and summarizes the parameter estimates from M4 at treatment group level; Fig. 2 displays all the predicted models against the observed data. From the segmented mixed model, the estimated changepoints and 95% confidence intervals for 150, 400 and 800 mg treatment groups were at 33.9 (28.7, 39.1), 57.4 (52.5, 62.4), and 52.8 (47.4, 58.1) hours after treatment, respectively. Counter to intuition, the lowest dose group (150 mg) showed the earliest changepoint, with nearly no clearance activity in the first phase with HRPC -0.8% (-2.5, 0.9%) and a fast clearance in the second phase 18.6% (16.0, 21.1%). For the other two treatment groups, the HRPC in the first phase were 400 mg: 0.9% (0.2, 1.6%), 800 mg: 1.9% (1.1, 2.6%); in the second phase, the HRPC were 400 mg: 16.8% (14.3, 19.1%) and 800 mg: 11.7% (9.3, 14.1%). To be noted, although the data is not shown in this manuscript, 3 out of 6 participants in the 150 mg treatment group, 2 out 8 participants in the 400 mg treatment group and 0 out of 8 in the 800 mg group developed recrudescence after the parasite count was not detected after M5717 dosing [11].

**Table 1:** Parasite clearance rate and changepoint estimates from M4, all methods

Method Treatment group	Parameter estimates with 95% CI				
	CP (95% CI, hours)	Slope before CP (log_10_[parasites/ml] / hour)	Slope after CP (log_10_[parasites/ml] / hour)	HRPC before CP (%)	HRPC after CP (%)
Segmented mixed model					
150 mg (n = 6)	33.9 (28.7, 39.1)	0.003 ( − 0.004, 0.011)	− 0.090 (− 0.103, − 0.076)	− 0.8 (− 2.5, 0.9)	18.6 (16.0, 21.1)
400 mg (n = 8)	57.4 (52.5, 62.4)	− 0.004 (− 0.007, − 0.001)	− 0.080 (− 0.092, − 0.067)	0.9 (0.2, 1.6)	16.8 (14.3, 19.1)
800 mg (n = 8)	52.8 (47.4, 58.1)	− 0.008 (− 0.011, − 0.005)	− 0.054 (− 0.066, − 0.042)	1.9 (1.1, 2.6)	11.7 (9.3, 14.1)
Grid − Search Segmented mixed model					
150 mg (n = 6)	35 (NA)	0.000 (− 0.006, 0.006)	− 0.088 (− 0.099, − 0.076)	− 0.0 (− 1.4, 1.3)	18.3 (16.1, 20.4)
400 mg (n = 8)	54 (NA)	− 0.003 (− 0.006, 0.001)	− 0.072 (− 0.082, − 0.062)	0.6 (− 0.2, 1.4)	15.3 (13.3, 17.2)
800 mg (n = 8)	52 (NA)	− 0.008 (− 0.012, − 0.005)	− 0.053 (− 0.063, − 0.043)	1.9 (1.1, 2.6)	11.5 (9.5, 13.6)
Two-stage segmented regression approach					
150 mg (n = 6)	36.1 (29.9, 42.2)	0.000 (− 0.006, 0.006)	− 0.093 (− 0.107, − 0.078)	0.1 (− 1.3, 1.4)	19.2 (16.4, 21.9)
400 mg (n = 8)	58.9 (53.2, 64.6)	− 0.004 (− 0.007, − 0.001)	− 0.081 (− 0.094, − 0.068)	0.9 (0.3, 1.6)	17.0 (14.4, 19.5)
800 mg (n = 8)	51.8 (45.8, 57.7)	− 0.008 (− 0.011, − 0.005)	− 0.052 (− 0.065, − 0.039)	1.8 (1.2, 2.4)	11.3 (8.7, 13.8)

*CP* changepoint, *CI* confidence interval, *HPRC* hourly rate of parasite clearance

The results from the grid search methods based on M4 and meta-analysis are also summarized in Table 1. The selected changepoints point estimates from the grid search method did not have precision estimates because of its non-parametric nature. The final models from all three methods demonstrated a strong fit to the data (Fig. 2). In general, the results from all three methods were similar, as demonstrated by the fitted models from the three methods mostly being overlapping. One exception was participant 8 in the 800 mg treatment group whose individual level model from the two-stage approach appeared different from the two other methods. This individual’s baseline parasitaemia was lower than required by the study protocol and the fluctuation before hour 50 made fitting individual level model difficult/unreliable. Nevertheless, the segmented mixed effects models were able to absorb this deviation and fit models through the observed data points.

## Discussion

The segmented mixed model with changepoint as a parameter estimated using the likelihood-based method [17], was shown to be a convenient one-step tool to characterize parasitaemia clearance data with multiple phases of linear pattern. All parameters of interest including slopes and changepoints can be modelled with random effects and as functions of covariates. Precision estimates for these parameters are either provided directly or can be derived. There are many features of Muggeo’s method that make it suitable for this analysis. Firstly, by allowing random effects for intercept, slopes and changepoint, the segmented mixed model accounts for within participant correlation due to repeated measurements. Secondly, the segmented mixed model allows for two (or more) slopes: e.g. $\beta_{1}$ for slope 1, $\beta_{1} + \delta$ for slope 2. Thirdly, the intercept, slopes and changepoint can be functions of covariates, which enables estimation of the slopes and the changepoint for each treatment group by including treatment group as a covariate. Fourthly, as a pooled analysis and a contrast to the two-stage approach, the model uses data efficiently and is robust to outlying data points or individuals. Fifthly, the model provides fit statistics e.g. AIC and BIC which facilitate comparison between models with and without any covariates or random effects and interactions terms. In addition, the same as the other methods studied, the model does not require all participants to have the same measurement frequency or the same number of measurements.

The segmented mixed model with grid search for changepoint compares model fit given different possible values. The selected changepoints, the HRPC estimates and confidence intervals are similar to those estimated using likelihood-based method [21]. However, the grid-search method is computationally excessive. Due to the grid searching nature, one limitation of this approach is that only finite sets of possible changepoints can be chosen from. In this analysis, the changepoints were examined at integer values. Fractional values are possible, but to test them all is computationally intensive and unfeasible in practice.

Another limitation of the grid search method is that precision estimates of the selected changepoints are not available. One possible solution is bootstrapping. However, several properties of the data make it improper. Firstly, as applicable to all regression models, bootstrapping for this data would have to be based on entries instead of single values. The explanatory variables are fixed, and each round of resampling would lose some information. Secondly, bootstrapping requires data entries to be independent, however this data is on the opposite, i.e. repeated observations within participants. The resampling has to be done at participant level. When the sample size is small, i.e. n < 50, the reverse percentile based confidence intervals tend to be too narrow [25]. In this study, there were only 22 participants. Thirdly, bootstrapping would be computationally excessive, even if proper. One round of grid search is already computationally heavy, i.e. K $\times$ M $\times$ L segmented mixed effects models are usually in the thousands and bootstrapping for confidence intervals would require the whole process to be repeated thousands of times, meaning millions of models to be run. In short, bootstrapping for estimating the precision of the estimated changepoints was considered to be improper and very challenging, if at all possible.

The two-stage approach of fitting a segmented model for each participant followed by meta-analysis to estimate dose-specific estimates, had similar results to the one-step methods (Table 1, Fig. 2). There has been literature investigating the efficiency of using meta-analysis of summary results and joint analysis of individual participant data, which suggests that theoretically and numerically, meta-analysis of summary results is statistically as efficient as joint analysis of individual participant data [26, 27]. As expected, the results from the two-stage approach (i.e. analysis of individual level data followed by meta-analysis) were similar toa one-stage approach (i.e. overall mixed effects model). Although, as noted already, at individual level, the fit of segmented model could be problematic due to limited numbers of observations or measurements, although fortunately this was not the case for this data. The analysis performed for this study was mainly a methodology exploration of mixed effect segmented model approaches to estimate parasite clearance trajectory for the bi-phasic clearance observed in the M5717 phase I trial [11]. The results from the two-stage approach presented here uses similar methodology to the two-stage approach presented in the M5717 phase I publication [11]. The major difference, is that in this current study, all 22 participants were used in the meta-analysis for treatment group estimates, whereas in the M5717 phase I trial publication, three of the 22 participants were not. Minor differences between the analyses could be because different estimation/maximization options in statistical functions or different precisions were kept in intermediate calculation results. As discussed in the M5717 phase I publication [11] the mechanism underlying the biphasic clearance is likely related to the mode of action of M5717 in the process of protein synthesis and possibly also related to the qPCR technology used which does not differentiate between viable and dead parasites.

This research work has some limitations. There are other factors which could potentially impact the performance of the three methods but were not studied, such as sample size (number of individuals and number of observations in each), the quality and complexity of the data, the underlying mechanism for generating the data and the specific assumptions to be taken (e.g. biphasic or other mutiphasic, straight line in each phase and the mode of connection between different phases). Another limitation is that the analyses performed were restricted to this data-set, i.e. the 3 specific dose levels and repeated measurements from 0 to 144 h post-treatment. The interpretation of the models should stay within the data ranges and extrapolation beyond these ranges should be avoided.

## Conclusions

Segmented mixed model with random changepoints as estimated using a likelihood-based method [21], is a convenient one-step tool to characterize the bi-phasic parasite clearance of M5717 in the Phase 1b study. Alternative methods including segmented mixed effects models with grid search to estimate the changepoint and the two-stage approach by fitting a segmented regression model for each individual followed by meta-analysis yield similar estimated parasite clearance rates and changepoints for the three treatment groups. However, the segmented mixed model with random changepoints is a computationally and mathematically efficient approach as precision estimates are calculated for the changepoints, covariates can be included and it is more robust to outlying or influential data points.

## Supplementary Information

**Additional file 1: **Metrics for parasite clearance rate: hourly rate of parasite clearance (HRPC), parasite clearance half-life and parasite reduction ratio (.word file: details of HRPC).

**Additional file 2: **Model building based on segmented mixed models (.word file: details of the four models with different fixed and random effects).

**Additional file 3: **Fit of models based on segmented mixed effects model with parametric changepoint (word file: detail showing model M4 had best fit to the data).

## Data Availability

Any requests for data by qualified scientific and medical researchers for legitimate research purposes will be subject to Merck KGaA, Darmstadt, Data Sharing Policy. All requests should be submitted in writing to Merck’s data-sharing portal https://www.merckgroup.com/en/research/our-approach-to-research-and-development/healthcare/clinical-trials/commitment-responsible-data-sharing.html. When Merck KGaA, Darmstadt, has a co-research, co-development, or co-marketing or co-promotion agreement, or when the product has been out-licensed, the responsibility for disclosure might be dependent on the agreement between parties. Under these circumstances, Merck KGaA, Darmstadt, will endeavour to gain agreement to share data in response to requests.
