# Supplementary material for: Evaluation of a multiphasic parasite clearance profile after treatment of experimental human infection with the investigational anti-malarial M5717 using segmented mixed effect models
Source: Malar J. 2023 Jun 28;22:199. doi: 10.1186/s12936-023-04627-x (PMC10303793; doi:10.1186/s12936-023-04627-x)
Supplement: Supplementary file 1 — Additional file 1: Metrics for parasite clearance rate: hourly rate of parasite clearance (HRPC), parasite clearance half-life and parasite reduction ratio (.word file: details of HRPC). [file 12936_2023_4627_MOESM1_ESM.docx]

**Additional file 1: Metrics for parasite clearance rate: hourly rate of parasite clearance (HRPC), parasite clearance half-life and parasite reduction ratio**

In this study the parasite clearance rate was depicted using hourly rate of parasite clearance (HRPC) which is the percentage of parasites cleared in each hour relative to the parasite level at the beginning of each hour. This term and other common measures such as parasite clearance half-life ${(t}_{1/2})$and parasite reduction ratio (PRR) are all derived from the regression coefficient ($slope)$ of $\log_{10} ($parasitemia/mL$)$ over time (per hour):

- HRPC=$1-{10}^{slope}$
- Parasite reduction ratio in 48 hours =${10}^{-48}\times slope$
- $t_{1/2}=\frac{{log}_{10}(2)}{-slope}$

With segmented models in which two or more slopes are estimated, these metrics for parasite clearance rates are all segment/phase specific.
