# Supplementary material for: Evaluation of a multiphasic parasite clearance profile after treatment of experimental human infection with the investigational anti-malarial M5717 using segmented mixed effect models
Source: Malar J. 2023 Jun 28;22:199. doi: 10.1186/s12936-023-04627-x (PMC10303793; doi:10.1186/s12936-023-04627-x)
Supplement: Supplementary file 2 — Additional file 2: Model building based on segmented mixed models (.word file: details of the four models with different fixed and random effects). [file 12936_2023_4627_MOESM2_ESM.docx]

**Additional file 2: Model building based on segmented mixed models**

The outcome variable is log_10_(parasites/mL). All models were adjusted for parasite levels at the time of treatment administration (baseline), and cohort (treatment group) assuming instant parasite level depends on the initial parasite level and treatment dose. Random intercept was included in all the models below. Treatment group (noted as *cohort* in the below models) was used as a categorical variable as the dose-response relationship was not assumed to be linear.

*Model M1: fixed and same slopes for all treatment groups, fixed and same changepoint for all treatment groups*

Fixed effect: *basep* ${(\beta}_{0})$, *cht1,* *cht2*, *time (*$\beta_{1})$, *U*$(\delta)$, *G0* $(\psi)$

Random effects: Intercept only

$$y_{ij}= \beta_{0i}+\beta_{1}t_{ij}+\delta\left( t_{ij}-\psi\right)I\left( t_{ij}>\psi\right)+\varepsilon_{ij}$$

where $\beta_{0i}= \beta_{0}+b_{0i}$, $b_{0i}\sim N\left( 0,\sigma_{b0}^{2} \right)$ and $\epsilon_{ij}\sim iid N\left( 0, \sigma_{\epsilon}^{2} \right)$,

and the parameters are defined in the Modelling Methods of the main manuscript.

*Model M2: fixed slopes but different for treatment groups, fixed changepoint but different for treatment groups*

Fixed effects: *basep*, *cht1*, *cht2*, *time*, *cht1:time*, *cht2:time*, *G0*, *G.cht1*, *G.cht2*, *U*, *U.cht1*, *U.cht2*

Random effects: *Intercept* only

*Model M3: random slopes, fixed changepoint but different for treatment groups*

Fixed effects: same as M2

Random effects: *Intercept*, *time*, *U*

*Model M4: random slopes and random changepoint*

Fixed effects: same as M2

Random effects: *Intercept*, *time*, *U*, *G0*

In these models, *basep* is the variable name for baseline parasite level, log10(# parasites) at hour 0; *cht1* and *cht2* are indicator variables for cohort 1 and cohort 2 respectively, with cohort 3 used as the reference; *time* is the time variable and its coefficient is the slope 1 for Cohort 3; *cht1:time* and *cht2:time* are the interactions between cohorts and time for slope 1, so that sum of coefficients for *time* and *cht1:time* is the slope 1 for Cohort1, and so on; *G0* is the parameter for changepoint, and *G0*, *G.cht1+G0* and *G.cht2+G0* are the changepoints for Cohorts 3, 1 and 2, respectively; *U* is the parameter for difference between slope 1 and slope 2, and *U*, *U.ch1+U* and *U.cht2+U* are the differences between slope 1 and slope 2 for Cohorts 3, 1 and 2, respectively. A point estimate and a standard error for each of these parameters are provided in the output.

In models M2, M3 and M4, the construction of point estimate and precision of slope 2 is slightly complex. In these models cohort 3 was used as the reference group. For cohort 3, the slope 2 estimate is the sum of the coefficient estimates for: *time* and *U*. For cohort 2, the slope 2 estimate is the sum of the coefficient estimates for: *time*, *cht2:time*, *U*, and *U.cht2*; similarly, the slope 2 for cohort 1 is the sum of the coefficients for: *time*, *cht1:time*, *U*, and *U.cht1*. The variance of slope 2 for each cohort can be derived from the variance-covariance matrix provided in the model output as a combination of variables, using the following formula:

$$Var\left( \sum_{l=1}^{m} X_{l} \right)=\sum_{l=1}^{m} Var(X_{l})+2\sum_{1\leq l<k\leq m} Cov(X_{l}{, X}_{k})$$

where $X_{l}$ is the $l^{th}$ parameter involved in the respective list.
