# Supplementary material for: Evaluation of a multiphasic parasite clearance profile after treatment of experimental human infection with the investigational anti-malarial M5717 using segmented mixed effect models
Source: Malar J. 2023 Jun 28;22:199. doi: 10.1186/s12936-023-04627-x (PMC10303793; doi:10.1186/s12936-023-04627-x)
Supplement: Supplementary file 3 — Additional file 3: Fit of models based on segmented mixed effects model with parametric changepoint (word file: detail showing model M4 had best fit to the data). [file 12936_2023_4627_MOESM3_ESM.docx]

**Additional file 3: Fit of models based on segmented mixed effects model with parametric changepoint**

The Akaike information criterion (AIC) statistics of the four tested models based on segmented mixed effects model with parametric changepoint (1) are summarized in the Table. M2 shows a considerably better fit than M1, indicating that the slopes and changepoint vary across treatment groups. Among M2, M3 and M4, M4 clearly shows the best fit to the data, which means random effects in slopes and changepoints are necessary.

| Model |  | Akaike information criterion |
| --- | --- | --- |
| M1 |  | 569 |
| M2 |  | 376 |
| M3 |  | 224 |
| M4 |  | 151 |

Model M1: fixed and same slopes for all treatment groups, fixed and same changepoint for all treatment groups; random effects for intercept only

Model M2: fixed slopes but different for treatment groups, fixed changepoint but different for treatment groups; random effects for intercept only

Model M3: random slopes, fixed changepoint but different for treatment groups; random effects for intercept and both slopes

Model M4: random effects for intercept, slopes and changepoint

**References**

1. Muggeo VMR, Atkins DC, Gallop RJ, Dimidjian S. Segmented mixed models with random changepoints: a maximum likelihood approach with application to treatment for depression study. Statistical Modelling. 2014;14(4):293-313.
